# The association between oral health and risk behaviours of university students

**DOI:** 10.1371/journal.pone.0309183

**Published:** 2025-03-18

**Authors:** Tanzeelah Azam, George Kitsaras, Juliana Gomez, Michaela Goodwin

**Affiliations:** 1 Dental Health Unit, University of Manchester, Manchester, United Kingdom; 2 Dental Health Unit, Colgate-Palmolive, Manchester, United Kingdom; Shahid Beheshti University of Medical Sciences School of Dentistry, IRAN, ISLAMIC REPUBLIC OF

## Abstract

**Background:**

Young adults are exposed to a variety of risk-related behaviours such as alcohol, smoking, and changes in dietary habits, which may result in unknown outcomes in their oral health. There is limited evidence on whether different risk behaviours are associated with oral health behaviours in the university student population. This study gathers data on the behaviours of students in their first year of university, which will inform the future development of oral health behaviour change interventions for this population.

**Method:**

This longitudinal quantitative survey involved 205 first-year students aged 18-24 at the University of Manchester. Students completed online questionnaires at baseline and again at a 6-month follow-up interval, providing information regarding self-reported oral health status, hygiene routines, and risk behaviours (e.g., diet, smoking, alcohol).

**Results:**

The findings showed associations between oral health behaviours with risk behaviours, including links with oral care routines, bleeding gums, brushing frequency, with exercise, vaping, and unhealthy food and drink intake. Significant changes over the two-time points were also observed, including the worsening of the self-reported condition of the teeth (p < 0.001), a reduction in the self-reported condition of the gums (p = 0.004), a decrease in brushing frequency (p = 0.003), fewer regular dental visits (p = 0.013), more students intending to visit their previous dentist rather than finding a new dentist at university (p = 0.026), and greater consumption of unhealthy non-alcoholic drinks (p = 0.003). Positive changes over time included reduced frequency and units of alcohol consumption (p = 0.030 and p = 0.001), fewer instances of binge drinking (p = 0.014), and less frequent consumption of unhealthy foods (p = 0.034).

**Conclusion:**

The findings highlighted the complex relationship between oral health and risk behaviours in this demographic. Poorer oral health behaviours were linked to engagement in risk behaviours. Thus, oral health behaviours should be targeted alongside other risk behaviours, and tailored interventions should be developed to improve behaviours among university students.

## Introduction

Oral health plays a significant role in overall general health and wellbeing [1]. Poor oral health is also associated with many diseases such as heart disease, stroke [2], atherosclerosis [3], diabetes [4], and cognitive decline over time [5]. Poor oral health practices can lead to oral diseases, such as dental caries and gum disease which result in pain and discomfort [6], impact an individual’s ability to consume foods, communicate with others, and influence their overall state of well-being [7,8]. Oral diseases can also negatively affect the quality of life [9], affect academic performance [10] and lead to time off university for treatment and pain [11]. It is therefore crucial to ensure that the oral health of students is understood and addressed.

Young adults in university are exposed to new experiences during their time of transition from teenagers to adults [12]; this exposure may lead to changes in lifestyle choices which involve engaging in risk-related behaviours such as alcohol consumption, smoking, poor eating habits, poor diet, and lack of exercise [13]. These behaviours adopted during young adulthood can persist into later stages in life, potentially leading to future health issues [14]. Moreover, while evidence exists regarding the dental health of young adults, the university setting introduces a period of newfound independence and exposure to various risk behaviours [15]. These behaviours can negatively affect individuals [13]. Consequently, the overall habits of university students may change, potentially exacerbating existing habits and behaviours. Recognising the tendency of university students to adopt unhealthy behaviours is important [16, 17], as it becomes more difficult to promote behaviour change as individuals get older [18].

This area of research remained underexplored, with limited evidence available to comprehensively understand the correlation between oral health behaviours and risk behaviours in this demographic, particularly during the key life transition of leaving a family home for the first time and gaining greater independence. Some evidence suggests that individuals in this population are exposed to risk behaviours during this period, however, there is a need for further investigation of the links between these oral health and risk behaviours, observing behaviours that occur over the first year of this life event, and the overall interplay with oral health behaviours [19]. Our initial patient and public involvement (PPI) work [20] indicated that university students considered oral health important and were open to participating in research that required sharing insights into their oral health behaviours and risk behaviours.

Overall, this study addressed evidence gaps and aimed to build evidence on the profiles of this population regarding oral health and risk behaviours. This study successfully achieved its aim by providing valuable insights and identifying areas for targeted intervention. Developing an oral health intervention tailored to this demographic would require a deeper understanding of the links to facilitate positive behaviour change.

This study aimed to evaluate self-reported oral health, dental attendance, oral health behaviours, and health-related risk behaviours in university students, tracking changes or stability over time as they transitioned to living independently. This aim was achieved through the following objectives: employing a quantitative survey within a university student population to gather self-reported data describing: (a) oral health status and dental attendance, (b) oral health behaviours, and (c) oral and general health-related risk behaviours.

## Methodology

The study utilised a prospective cohort design with a longitudinal, quantitative survey conducted over 6-months.

### Recruitment

The recruitment process followed a convenience sampling approach [21] using advertisements on the University of Manchester research volunteering website, posters in university buildings, and university social media pages to attract participants. An initial sample size consisted of 205 participants, with comparisons between the two time points involving 114 students. A total of 205 participants volunteered and were recruited for the study. Screening of participants was based on the eligibility inclusion criteria, which required participants to be: (a) be between 18-24 years old, (b) be in their first year of university (c) be studying or attending university for the first time, (d) live away from their family home, (e) have access to a computer or mobile phone with internet connection, (f) be able to comprehend and speak English, (g) be available and attending campus throughout the duration of the study, and (h) have completed an informed written consent form. The recruitment period began on 1^st^ October 2022 and ended on 31^st^ January 2023. The study design and protocol were approved by the University of Manchester Research Ethics Committee (Reference: 2023-13950-27733).

### Data collection

The study gathered data through online questionnaires administered via Qualtrics software [22]. The questionnaires were customised specifically for the university student population, based on prior patient and public involvement (PPI) work to improve the relevance and accuracy of data collection. At baseline, participants completed a demographic questionnaire (S1 Table) to capture basic participant information, and a behaviour questionnaire (S2 Table) designed to assess oral health and risk behaviours. At six months follow-up, participants were asked to complete the behaviour questionnaire again, along with a feedback questionnaire to gather participants’ perceptions and experiences of the study. The data collection points were pre-determined, with baseline data gathered at the start of the study and follow-up data collected exactly six months later.

Data was analysed descriptively, (using frequencies, counts, and trends) to assess changes over time. SPSS software [23] was used to conduct statistical analysis. Chi-squared tests examined associations between categorical variables across groups, with sub-grouping adjustments made if any expected counts were below 1. In cases where this criterion were unmet, Fisher’s Exact test was employed. For longitudinal analysis, the McNemar test analysed changes in dichotomous variables within groups over time (baseline vs follow-up), while the Wilcoxon Signed-Rank test was applied for continuous and/or ordinal variables. The significance level for all tests was set at p < 0.05.

## Results

### Demographics of sampling characteristics

Excluding those who did not meet the inclusion criteria, 290 participants consented to participate in the study. Of these, 205 completed the study, yielding a response rate of 70.7%, with no dropouts at baseline. Participants consisted of 66% female (n = 135) and 34% male (n = 70). Missing data appeared at random, with no discernible patterns and the mean age did not significantly differ between baseline and follow-up. Participants were ethnically diverse: 52.2% were White, 32.2% Asian or British Asian and, 5.4% Black, Black British, Caribbean, or African. At follow-up, 116 participants completed data collection, resulting in a response rate of 56.6%, with 89 dropping out from baseline. After excluding two participants with incorrect ID numbers, 114 completed data collection. The gender ratio at follow-up was 68.4% female (n = 78) and 31.6% male (n = 36). The ethnicity distribution remained similar to baseline, with 46.5% White, 32.5% Asian or British Asian and, 7.0% Black, Black British, Caribbean, or African.

### Oral health behaviours

At baseline, oral health behaviours showed that students considered their oral care routine and the condition of the teeth and gums as average to good. Approximately half of students prioritised fresh teeth and clean teeth (58.0% and 49.1%) as the most important aspects regarding their mouth. Of the 114 students, 70.2% reported brushing twice a day, while 32.5% had experienced toothache during the study. Just over half of students reported that their gums did not bleed when brushing (60.5%). In terms of dental visits, a notable portion of responses indicated students had visited the dentist less than 6 months ago (29.8%), primarily for a routine check-up (63.2%) with a smaller proportion attending for treatment such as a filling or extraction (15.8%). However, 95.6% of students had not registered with a dentist since starting university, with 60.5% intending to continue visiting their current dentist.

At follow-up, students prioritised having white teeth and avoiding toothache (50.9% and 28.1%) as the most important aspects regarding their mouths. A total of 68.4% of students reported brushing their teeth twice a day, with levels of bleeding gums remaining unchanged. Regarding dental visits, 32.5% of students visited the dentist more than 1 year ago but less than 2 years ago for similar reasons as at baseline. Students continued to not register with a new dentist (95.6%) and a higher proportion of students intended to continue visiting their current dentist (78.1%). All data is available in S3 Table.

### Risk behaviours

Baseline data on risk behaviours revealed that 10.5% of students smoked, predominantly less than 5 cigarettes daily (83.3%), and started 6-12 months ago (50.0%). Vaping was less common, with 75.4% having never vaped and 9.6% doing so. A total of 67.5% of students drank alcohol, typically 2–4 times monthly or 2–4 times a week (both 32.5%), and would commonly consume between 3–4 units or 5–6 units per day (both 28.6%), though consumption of 8 or more units varied. A large portion of students exercised (75.6%), with 38.6% engaging more than once a week, primarily carrying out general fitness or were at the gym (43.0%). Food intake was rated as average by 54.4% of students, with high consumption of sugary items like biscuits, cakes, cream cakes, and sweet pies (86.8%), often consumed several times a week (46.5%). Non-alcoholic drink intake was also believed to be average according to the participant responses from the Likert scale, with 87.5% consuming such drinks 1-2 times daily, such as sugary soft drinks (52.6%). Some students consumed energy drinks (22.8%), with 88.5% reporting changes in consumption since starting university.

The follow-up data on risk behaviours indicated that 7.0% of students smoke, with 75.0% smoking fewer than 5 cigarettes daily. Half of students who smoked reported they began smoking over a year ago but less than two years ago (50.0%). Regarding vaping, 11.4% of students vaped, with 69.3% having never done so before. Over half of students drank alcohol (57.0%), with 43.1% drinking monthly or less, and typically consuming 1–2 units (41.5%). The frequency of consuming 8 or more units varied but was generally lower levels than baseline. Exercise frequency remained high, with 56.1% engaging at least once a week, often through activities such as walking or going to the gym. On a Likert scale, ranging from very unhealthy to very healthy, food intake was rated as average or healthy (43.0% for both) with high consumption of sugary items (86.0%), often consumed several times weekly (45.6%). Non-alcoholic drink intake was considered average by 40.4% of students, according to participant responses from the Likert scale, and 68.4% of students consumed such drinks 1–2 times daily, with a preference for sugary drinks like Cola (61.4%). Approximately one-third of students drank energy drinks, primarily 1–2 per day, with 94.4% of students reporting changes in drinking habits since starting university. Baseline and follow-up comparisons were made only with students who completed both questionnaires at baseline and follow-up. All data is available in S4 Table.

### Significant study data

There were significant associations between different variables in the study, including oral health behaviours and risk behaviours. At baseline, the associations that were observed are shown in Table 1 and the associations at follow-up are shown in Table 2.

**Table 1:** Associations between different variables in the study behaviour data at baseline.

Study data comparisons (baseline):	Direction of association	Test statistic^a^, P value^b^, Effect size^c^
Exercise and oral care routine rating	Exercising more frequently associated with a better oral care routine rating	X^2^ (8) = 21.065, p = 0.008, Cramer’s V = 0.227
Food intake and condition of the gums	Unhealthy food consumption associated with poorer self-reported condition of gums	X^2^(6) = 16.786, p = 0.010, Cramer’s V = 0.202
Frequency of food intake and condition of the teeth	Consuming unhealthy foods more frequently associated with less healthy teeth	X^2^(6) = 22.414, p = 0.001, Cramer’s V = 0.234
Non-alcoholic drink intake and oral care routine rating	Healthy non-alcoholic drink intake associated with better oral care routine	X^2^(6) = 16.890, p = 0.010, Cramer’s V = 0.203

^a^Chi-squared test with degrees of freedom.

^b^Significance level is set at p < 0.05.

^c^Cramer’s V value.

**Table 2:** Associations between different variables in the study behaviour data at follow-up.

Study data comparisons (follow-up)	Direction of association	Test statistic^a^, P value^b^, Effect size^c^
Exercise and bleeding gums	Exercising less frequently associated with bleeding gums	X^2^(4) = 13.113, p = 0.011, Cramer’s V = 0.339
Exercise and brushing frequency	Exercising more frequently associated with brushing more frequently	X^2^(2) = 8.822, p = 0.012, Cramer’s V = 0.278
Vaping status and bleeding gums	Never vaped associated with no bleeding gums	X^2^(2) = 8.122, p = 0.017, Cramer’s V = 0.267
Non-alcoholic drink intake and oral care routine rating	Healthy non-alcoholic drink intake associated with better oral care routine	X^2^(4) = 17.030, p = 0.002, Cramer’s V = 0.273
Non-alcoholic drink intake and condition of gums	Healthy non-alcoholic drink intake associated with better self-reported condition of gums	X^2^(6) = 20.935, p = 0.002, Cramer’s V = 0.303
Frequency of non-alcoholic drink intake and condition of the teeth	Less frequent non-alcoholic drink intake associated with better condition of teeth	X^2^(6) = 19.797, p = 0.003, Cramer’s V = 0.295
Frequency of non-alcoholic drink intake and brushing frequency	More frequent non-alcoholic drink intake associated with brushing less frequently	X^2^(6) = 24.637, p < 0.001, Cramer’s V = 0.329

^a^Chi-squared test with degrees of freedom.

^b^Significance level is set at p < 0.05.

^c^Cramer’s V value

At both baseline and follow-up, various different associations were observed between risk behaviours and oral health status. The data indicated some associations between increased risk behaviours and poor oral health. For instance, unhealthy food consumption was associated with a self-reported poorer condition of the gums, and increased intake of non-alcoholic drinks correlated with less frequent tooth brushing. Conversely, the data also indicated associations between reduced engagement in risk behaviours and improved oral health. Examples include findings where students who reported never vaping also reported no bleeding gums, and those who consumed healthier non-alcoholic drinks felt they had a better oral care routine.

### Comparisons between baseline and follow-up

Associations between variables in the study data at the two time-points (baseline and follow-up) are shown in Table 3.

**Table 3:** Associations between variables in the study data across the two time-points.

Comparisons significances over time points:	Test statistic^a^, P value^b^, Effect size^c^
Condition of the teeth	Z = -3.499, p < 0.001, r = -0.32
Condition of the gums	Z = -2.873, p = 0.004, r = -0.27
Brushing frequency	Z = -2.987, p = 0.003, r = -0.27
How long since last visit to the dentist	Z = -2.497, p = 0.013, r = -0.23
Intend to visit home dentist or new dentist	Z = -3.035, p = 0.002, r = -0.28
Alcohol consumption frequency	Z = -2.169, p = 0.030, r = -0.20
How many units of alcohol	Z = -5.651, p < 0.001, r = -0.53
8 or more units of alcohol	Z = -2.450, p = 0.014, r = -0.23
Frequency of food intake	Z = -2.903, p = 0.004, r = -0.27
Non-alcoholic drink intake	Z = -2.083, p = 0.037, r = -0.20

^a^Wilcoxon signed-rank test,

^b^Significance level is set at p < 0.05,

^c^Correlation coefficient r statistic, refers to an increase over study period, refers to a decrease over study period

Overall, many patterns and trends emerged in the data, for the oral health behaviours and risk behaviours in this demographic. Some oral health behaviours showed no statistical differences over time, including the rating of oral care routine (p = 0.075), bleeding gums (p = 0.567), and toothache (p = 0.410). Additionally, several risk behaviours showed no statistically significant changes over time such as smoking (p = 0.401), vaping (p = 0.431), weight satisfaction (p = 0.488), energy drink consumption (p = 0.253), frequency of energy drink intake (p = 0.194), and changes in energy drinks consumption (p = 0.105). Exercise levels also remained stable (p = 0.976). In relation to dental attendance, the importance of placed on regular visits and reasons for visiting the dentist showed no significant change over time (p = 0.324 and p = 0.687, respectively). More detailed non-significant comparisons can be seen in S5 Table. In summary, oral health behaviours, risk behaviour, and changes in behaviours over six months were explored, revealing associations between poor oral health behaviours and some risk behaviours, while certain behaviours remained unchanged since the start of university.

## Discussion

### Summary of findings

The use of surveys across a period of six months enabled a longitudinal evaluation of behaviours outlined in the objectives. The study revealed a comprehensive association between various lifestyle and oral health outcomes among university students. Specifically, alcohol consumption, vaping, and regular consumption of unhealthy drinks were all correlated with self-reported oral health, such as the deteriorating condition of the teeth, rating of oral care routine, and bleeding gums. Infrequent dental visits were prevalent among university students. However, a participants overweight status did not show a significant association with poor oral health, while exercise remains widespread within this demographic. Importantly, the study also highlighted an overall reduction in brushing frequency among university students. Together, the data gathered served to assess oral health behaviours and risk behaviours within the university student population.

The data revealed that at baseline, students prioritised having ‘fresh’ or ‘clean’ teeth, while at follow-up, their priorities shifted to having ‘white teeth.’ This shift suggested that students’ concerned evolved over time, possibly due to increased appearance-related concerns. Research supports these findings, as the appearance of teeth impacts an individual’s attractiveness and plays a role in social interactions [24,25]. Given that young adults are often more conscious of their appearance, especially in social settings [26], may have contributed to this desire for whiter teeth [27]. This highlights the importance of social interactions during a student’s time at university [28] and how dental appearance shapes perceptions and interactions [29].

The study findings raised awareness of the lack of registration for new dental services, with students intending to visit their previous dentist rather than finding a new one while at university. This may be due to the limited availability of National Health Service (NHS) dentists accepting new patients due to capacity issues [30]. Additionally, the timing of students’ last dental visits shifted from ‘less than 6 months ago’ to ‘more than 1 year ago but less than 2 years ago,’ indicating that students had not visited the dentist while at university at these time points. Further research is needed to explore whether students attend dental check-ups during their university years, as typical university programmes last 3-5 years while NHS dentist registration expires after two years [31,32]. This could result in many students not having an NHS dentist upon graduating university.

The rating of oral care routines was influenced by numerous factors, as shown by correlations with exercise and non-alcoholic drink intake. These associations demonstrated the interconnectedness of oral health behaviours with broader lifestyle choices. Research supports the idea that regular exercise can safeguard against diseases and promote overall well-being [33], including oral health benefits such as preventing periodontal disease [34]. This study also assessed the links between exercise and brushing frequency, demonstrating that students who are more physically active are more likely to brush twice a day, a finding supported by previous research [35]. Moreover, a deterioration in students’ oral health over time is evident, with worsening of self-reported conditions of the teeth and gums. The prevalence of bleeding gums among university students aligns with existing research findings [36]. An association between bleeding gums and vaping gums also emerged from the data, suggesting potential long-term implications of vaping on gum health, as supported by prior research [37–39].

The study findings indicated a decline in the number of students brushing twice a day and an increase in those brushing once a day (Z = -2.987, p = 0.003). This aligned with previous findings by Bashiru and Anthony 2014, who reported that 90% of students brushed their teeth once a day [40]. Possible reasons for the decreased brushing frequency included busy schedules, lack of routines, and late nights spent studying or socialising during their time at university. It could be speculated that factors such as increased workload, stress, or changes in routine may have contributed to this trend. As it has been shown that there is a correlation between brushing frequency, neglecting toothbrushing, and stress [41].

In this study, unhealthy food and drink consumption was associated with the condition of the gums, and dietary habits were shown to potentially impact oral health detrimentally, increasing the risk of gum disease [42,43]. The frequency of unhealthy dietary food and drink consumption was also linked to the condition of the teeth, indicating that poor dietary habits could negatively impact oral health outcomes. Over time, a decline in the frequency of unhealthy food consumption indicated a positive shift towards a more balanced dietary pattern. This transition suggested potential benefits for dental health by reducing the risk of tooth decay and dental issues [44]. By decreasing the frequency of sugary and unhealthy foods intake, students can minimise their teeth’s exposure to sugars and acids, thereby reducing the likelihood of enamel demineralisation, tooth weakening, erosion, and cavities [45]. Thus, healthy dietary habits are crucial to prevent tooth decay [46] and by cutting back on consumption, students can improve their oral health.

Alcohol consumption over time decreased overall, with reductions in the amount consumed, fewer units per occasion, and a decrease in binge drinking. Existing evidence supports this notion as students drink more alcohol at the start of university [47]. The trends in non-alcoholic drink intake among students revealed a prevalent consumption of sugary beverages, despite students perceiving their intake as average. This pattern suggests a normalisation of sugary drink consumption among students. Moreover, the findings suggested a connection between non-alcoholic drink intake and both the condition of the teeth and oral care routines, as individuals who consume unhealthy foods may drink more sugary beverages. This raised concerns about potential implications for dental health such as tooth decay and erosion [48,49]. In addition, 31.6% of students reported consuming energy drinks, an increase over time, with 94.4% noting a change in consumption since starting university. This trend aligned with prior studies on energy drink consumption among students. Possible motivations for this trend include improving academic performance, combating fatigue, socialising, and enjoying leisure time with friends [50].

### Key results and links

Data analysis revealed changes over time: (a) a self-reported deterioration of the condition of teeth and gums over time, (b) a shift in priority from fresh and clean teeth at baseline to the avoidance of toothache and having white teeth at follow-up, (c) a reduction in brushing frequency over time, (d) consistent levels of toothache across both time points but a general prevalence of toothache overall, (e) less frequent visits to the dentist, (f) check-ups and treatments (such as fillings and/or extractions) being the most common reason for dental visits, (g) a lack of students registering with new dentists, (h) a visible emphasis on the importance of attending the dentist, (i) an increase in students intending to visit their previous dentists, (j) general high levels of alcohol consumption, (k) a decrease in frequency of alcohol consumption over time, (l) a reduction in the number of units of alcohol consumed over time, (m) a decrease over time in the number of students consuming 8 or more units of alcohol on one occasion (binge drinking), (n) a general belief that smoking, alcohol, and vaping impacts oral health, (o) a consensus of perceived average, (p) consistently high exercise levels over time, (q) an unhealthier food intake at baseline with a reduction in unhealthy food consumption over time, (r) unhealthy non-alcoholic drink intake but improvements over time, (s) consistently high energy drink consumption over time, and (t) an increase in energy drink consumption since starting university.

### Direction of future work

Future research could focus on developing interventions specifically designed for university students aimed at addressing improving attitudes, oral health behaviours and associated risk behaviours prevalent during their academic period.

### Strengths

Feedback from participants, collected through the end-of-study questionnaire, reflected high levels of satisfaction and enjoyment, with 91.1% reporting enjoyment in participation, and 96.5% agreeing on the importance of the study topic. These results underscored strong support for the study. Participants’ preference for the online questionnaire format suggested their comfort and familiarity with digital platforms, reflecting frequent engagement with online tools, and highlighting the potential to leverage digital technology for future research endeavours or interventions. The study participants represented eight of the most enrolled-in degree programmes at the University of Manchester [51]. The use of a diverse sample with students enrolled in a wide range of academic subjects constitutes a strength of this study. By including participants from various fields, the research encompassed a broad spectrum of perceptive, enhancing the richness, and representativeness of the data collected. Importantly, this approach facilitated the capture of insights from individuals not exclusively studying health-related subjects, who might be more health conscious.

### Limitations

The reliance on self-reported data for assessing behaviours and attitudes may have introduced potential limitations due to social desirability and recall biases, as participants may have provided responses aligning to societal norms rather than accurately reflecting their behaviours. Efforts were made to mitigate this bias through confidentiality assurances. The anonymity provided by online questionnaires likely reduced potential social pressures or biases that could have arisen in face-to-face interactions. Therefore, leveraging digital platforms for data collection not only maximised participant engagement but also ensured a more efficient and inclusive process. Additionally, participant dropout from baseline to the six month follow-up led to a smaller-than-expected sample size, meaning that data from participants who only completed the baseline questionnaire could not be included in the comparative analysis between the two time periods. This attrition may have introduced potential attrition bias; however, the characteristics of the participants who completed the study showed no apparent differences from those who dropped out.

## Conclusion

The findings provided a comprehensive insight into the complex network of behaviours and attitudes among university students, revealing the prevalence of detrimental oral health behaviours and associated risk behaviours. Health outcomes and behaviours such as oral care routine rating, condition of the teeth, condition of the gums, bleeding gums, brushing frequency, exercise, vaping, and unhealthy dietary habits were found to be interconnected, indicating a complex interplay between various lifestyle choices. The study highlighted the critical period of university life, during which unhealthy behaviours often emerge and potentially solidify into lifelong habits. The challenges students faced in finding a new dentist and the lack of visits to the dentist during their time at university was concerning, highlighting the need for further initiatives aimed at promoting dental attendance among university students while they are residing away from home.

## Supporting information

S1 Table Demographic questionnaire. (DOCX)

S2 Table Behaviour questionnaire. (DOCX)

S3 Table Oral health behaviour percentages at baseline and follow-up. (DOCX)

S4 Table Risk behaviours percentages at baseline and follow-up. (DOCX)

S5 Table Non-significant associations observed from the behaviour questionnaire over time. (DOCX)
