# Supplementary material for: The association between oral health and risk behaviours of university students
Source: PLoS One. 2025 Mar 18;20(3):e0309183. doi: 10.1371/journal.pone.0309183 (PMC11918317; doi:10.1371/journal.pone.0309183)
Supplement: S1 Table — (DOCX) [file pone.0309183.s001.docx]

**Supporting information:**

**S1 Table: Demographic questionnaire**

| **Demographic questionnaire** | |
| --- | --- |
| Age |  |
| What is your gender? |  |
| What is your ethnicity? | White  Asian or Asian British  Black, Black British, Caribbean, or African  Mixed or multiple ethnic groups  Other ethnic groups  Other, please specify |
| What is the highest degree or level of school you have completed? | High school or equivalent  College, no degree  Bachelor’s degree (e.g. BSc, BA, BDS)  Master’s degree (e.g. MSc, MA)  Doctorate or professional degree (e.g. PhD) |
| Subject of study? |  |
