# Supplementary material for: The association between oral health and risk behaviours of university students
Source: PLoS One. 2025 Mar 18;20(3):e0309183. doi: 10.1371/journal.pone.0309183 (PMC11918317; doi:10.1371/journal.pone.0309183)
Supplement: S2 Table — (DOCX) [file pone.0309183.s002.docx]

**Supporting information:**

**S2 Table: Behaviour questionnaire**

| **Oral health related behaviours:** | | |
| --- | --- | --- |
| 1 | How would you rate your oral care routine (i.e. brushing teeth, flossing etc.) | 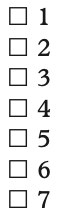Very good  Good  Average  Poor  Very poor |
| 2 | How would you describe the condition of your teeth? | Excellent  Very good  Good  Average  Poor  Very poor  Don’t know |
| 3 | How would you describe the condition of your gums? | Excellent  Very good  Good  Average  Poor  Very poor  Don’t know |
| 4 | What do you feel matters to you as a university student with regards to your teeth/mouth?  (Select all that apply) | Fresh breath  Having white teeth  Having straight teeth  Having clean teeth  Avoiding fillings  Avoiding tooth ache  Avoiding sensitivity  Avoiding bleeding gums |
| 4.a. | From the previous question, which of those options are your top two priorities or concerns? |  |
| 5 | On average, how many times per day do you normally brush your teeth? Please score as appropriate. | Less than once daily  Once daily  Twice daily  More than twice daily |
| 6 | Do your gums bleed when you brush your teeth? | Yes, always  Yes, sometimes  No, never |
| 7 | Have you experienced tooth ache in the past 6 months? | Yes  No |
| 8 | Do you use a toothpaste containing fluoride? | Yes  No  Don’t know |
| 9 | Do you use any of the following to clean your teeth?  (Select all that apply) | Toothbrush  Dental floss  Interdental brushes  Wooden toothpick  Chewstick/miswak  Mouthwash/rinse  Other (please specify below)  ………………………………………. |
| 10 | How long is it since you last saw a dentist? | Less than 6 months  6-12 months  More than 1 year but less than 2 years  More than 2 years but less than 5 years  5 years or more  Never received dental care |
| 11 | What was the reason of your last visit to the dentist? | Routine check-up  Pain or trouble with teeth/gums  Treatment - fillings or extractions  Treatment - orthodontic (braces)  Treatment - other (please specify below)  ........................................  Don’t know/don’t remember |
| 12 | Have you registered with a new dentist since starting university? | Yes  No |
| 13 | Do you feel it is important to attend the dentist regularly? | Yes  No |
| 14 | Do you intend to continue visiting your current dentist at home or find a new dentist? | Intend to continue attending home dentist  Intend to find a new dentist  Don’t know yet |
| **Risk behaviours** | | |
| 15 | What is your smoking status? | Currently smoking  Ex-smoker  Never smoked |
| 15.a. | If yes on smoking status, how many cigarettes do you smoke a day? Please rate as follows. | Less than 5  5-10  11-15  16-20  More than 20 |
| 15.b. | When did you start smoking? | Less than 6 months ago  6-12 months ago  More than 1 year but less than 2 years ago  More than 2 years but less than 5 years ago  5 years or more |
| 16 | Do you use any of the following types of tobacco? (Select all that apply) | Cigars  Chewing tobacco  Snuff  Other (please specify below) ………………………………  Not applicable |
| 17 | Do you vape? | Yes, I currently vape  No, but I used to vape  No, I have never vaped |
| 18 | Do you drink alcohol? | Yes  No |
| 18.a. | If yes to the previous questions, how often do you have a drink containing alcohol? | Never/rarely  Monthly or less  2-4 times a month  2-3 times a week  4 or more times a week |
| 18.b. | How many units of alcohol do you drink on a typical day?  (Wine: 125ml glass = 1.5 units, 175ml glass = 2.0 units; 250ml glass = 3units, pint of lower strength lager/beer/cider = 2 units; pint of higher strength lager/beer/cider = 3 units; single small shot of spirit = 1 1unit) | 1-2  3-4  5-6  7-9  10+ |
| 19 | How often do you have 8 or more units on one occasion? | Never/rarely  Less than monthly  Monthly  Weekly  Daily or almost |
| 20 | Do you think smoking, vaping or consuming alcohol affects the health of your mouth? (Please tick as many options as you like) | Smoking does  Vaping does  Alcohol does  No none of them do |
| 21 | How would you consider your weight? | Underweight  Average  Overweight  Obese |
| 21.a. | Are you happy with your weight? | Yes  No |
| 22 | Do you exercise? | Never/ rarely  Once a month  Once every 2/3 weeks  Once every week  More than once a week  If you exercise, please specify what form of exercise this is? (I.e., walking, jogging, gym)  ……………………………………………………………… |
| 23 | How would you consider your food intake? | Very healthy  Healthy  Average  Unhealthy  Very unhealthy |
| 23.a. | How would you consider your non-alcoholic drink intake? | Very healthy  Healthy  Average  Unhealthy  Very unhealthy |
| 24 | Do you eat any of the following foods, even in small quantities?  (Tick as many answers as you may) | Biscuits, cakes, cream cakes, sweet pies  Chewing gum containing sugar  Sweets/candy  Chocolate  Jam/honey/syrups  Take-aways  Sugary breakfast cereals  Not applicable |
| 24.a. | From the previous question, how often do you consume any of the foods? | Several times a day  Every day  Several times a week  Once a week  Several times a month  Seldom/never |
| 25 | Do you drink any of the following foods, even in small quantities?  (Tick as many answers as you may) | Soft drinks (e.g., Cola) with sugar  Soft drinks with no sugar (e.g. diet coke or coke zero)  Energy drinks  Milk with sugar  Tea with sugar  Coffee with sugar  Not applicable |
| 25.a. | From the previous question, how often do you consume any of the drinks per day? | 1-2  3-4  5+ |
| 26 | Do you drink energy drinks?  (e.g., Redbull/Monster) | Yes  No |
| 26.a. | If yes to the previous question, how many do you drink a day? | 1-2  3-4  5+ |
| 26.b. | Have you changed the amount and frequency of energy drinks you consume each day since starting university? | Yes  No |
