# Supplementary material for: The association between oral health and risk behaviours of university students
Source: PLoS One. 2025 Mar 18;20(3):e0309183. doi: 10.1371/journal.pone.0309183 (PMC11918317; doi:10.1371/journal.pone.0309183)
Supplement: S3 Table — (DOCX) [file pone.0309183.s003.docx]

**Supporting information:**

**S3 Table:** Oral health behaviour percentages at baseline and follow-up.

| **Oral health behaviours** | **Baseline** | **Follow-up** |
| --- | --- | --- |
| Oral care routine rating:  Average  Good  Very good  Poor | 48.2%  36.8%  11.4%  3.5% | 35.1%  49.1%  12.3%  3.5% |
| Self-reported condition of the teeth:  Good  Average  Very good  Excellent  Poor  Don’t know | 44.7%  26.3%  16.7%  6.1%  5.3%  0.9% | 39.5%  26.3%  20.2%  0.9%  12.3%  0.9% |
| Self-reported condition of the gums:  Good  Average  Very good  Excellent  Poor  Don’t know | 34.2%  31.6%  20.2%  6.1%  5.3%  2.6% | 22.8%  38.5%  24.6%  4.4%  7.9%  1.8% |
| Two top priorities: Fresh and clean teeth | 58.0% and 49.1% | 36.8% and 36.0% |
| Two top priorities: white teeth and avoiding toothache | 28.9% and 21.9% | 50.9% and 28.1% |
| Brush teeth twice daily  Brush teeth once daily  More than twice daily  Less than once daily | 70.2%  26.3%  2.6%  0.9% | 68.4%  28.9%  1.8%  0.9% |
| Experienced toothache | 32.5% | 38.6% |
| Used toothpaste to brush teeth | 100.0% | 100.0% |
| Gums do not bleed when brushed  Gums do bleed (sometimes or always) | 60.5%  39.5% | 59.6%  40.4% |
| Using toothpaste containing fluoride:  Yes  Don’t know  No | 64.9%  29.8%  5.3% | 71.1%  25.4%  3.5% |
| Using which products:   - Toothbrush - Mouthwash/rinse - Dental floss - Interdental brushes - Wooden toothpick - Chewstick/miswak - Other (coconut oil pulling) | 98.2%  43.0%  37.7%  8.0%  4.4%  0.9%  0.9% | 100.0%  36.8%  40.4%  8.8%  8.8%  0.0%  0.0% |
| Visited the dentist:  Less than 6 months  6-12 months  More than 1 year ago but less than 2 years ago  More than 2 years but less than 5 years  5 years or more | 29.8%  27.7%  22.8%  18.4%  1.8% | 25.4%  18.4%  32.5%  19.3%  4.4% |
| Reasons:   - Routine check-up - Treatment such as a filling or extraction - Pain or trouble with teeth/gums - Treatment – orthodontics (braces) - Other - Don’t know/don’t remember | 63.2%  15.8%  8.8%  7.0%  3.6%  1.8% | 64.9%  14.9%  9.6%  5.3%  3.4%  1.8% |
| Not registered with a dentist | 95.6% | 95.6% |
| Belief of importance of attending the dentist | 79.8% | 86.0% |
| Intend to continue visiting their current dentist  Intend to find a new dentist  Don’t know yet | 60.5%  17.5%  21.9% | 78.1%  8.8%  13.2% |
