# Supplementary material for: The association between oral health and risk behaviours of university students
Source: PLoS One. 2025 Mar 18;20(3):e0309183. doi: 10.1371/journal.pone.0309183 (PMC11918317; doi:10.1371/journal.pone.0309183)
Supplement: S4 Table — (DOCX) [file pone.0309183.s004.docx]

**Supporting information:**

**S4 Table:** Risk behaviours percentages at baseline and follow-up.

| **Risk behaviours** | **Baseline** | **Follow-up** |
| --- | --- | --- |
| Never smoked  Smoke  Ex-smoker | 84.2%  10.5%  5.3% | 86.8%  7.0%  6.1% |
| Consumption:  Less than 5 cigarettes daily  5-10 cigarettes daily | 83.3%  16.7% | 75.0%  25.0% |
| Consumption period:  Less than 6 months ago  Began smoking 6-12 months ago  More than 1 year ago but less than 2 years ago  More than 2 years ago but less than 5 years ago  5 years or more | 16.7%  50.0%  8.3%  25.0%  0.0% | 12.5%  12.5%  50.0%  25.0%  0.0% |
| Types of tobacco?  Cigarettes  Rolling tobacco  Not mentioned | 50.0%  8.3%  41.7% | 62.5%  12.5%  25.0% |
| Vaping status:  Never vaped  Currently vaping  Ex-vaper | 75.4%  9.6%  14.9% | 69.3%  11.4%  19.3% |
| Alcohol consumption | 67.5% | 57.0% |
| Consumption frequency:  2-4 times a month  2-4 times a week  Monthly or less  Never/rarely  4 or more times a week | 32.5%  32.5%  26.0%  5.2%  3.9% | 40.0%  16.9%  43.1%  0.0%  0.0% |
| Number of units per day:  1-2 units  3-4 units  5-6 units  7-9 units  10+ units | 18.2%  28.6%  28.6%  15.6%  9.1% | 41.5%  23.1%  21.5%  13.8%  0.0% |
| 8 or more units on one occasion frequency:  Never/rarely  Less than monthly  Monthly  Weekly  Daily or almost | 30.0%  27.3%  23.4%  18.2%  1.3% | 50.8%  23.1%  15.4%  10.8%  0.0% |
| Do you think smoking, vaping or consuming alcohol affects the health of the mouth?  Smoking, vaping and alcohol does  Smoking and vaping does  Smoking does  Smoking and alcohol does  No none of them do  Vaping does | 66.7%  14.9%  13.2%  3.5%  1.8%  0.0% | 60.5%  14.9%  6.1%  14.0%  3.5%  0.9% |
| How would you consider your weight?  Average  Overweight  Underweight  Obese | 80.7%  9.6%  8.8%  0.9% | 81.6%  10.5%  7.0%  0.9% |
| Are you happy with your weight?  No  Yes | 32.5%  67.5% | 27.2%  72.8% |
| Exercise:  More than once a week  Never/rarely  Once a week  Once a month  Once every 2/3 weeks | 38.6%  24.6%  16.7%  10.5%  9.6% | 40.4%  21.1%  15.8%  12.3%  10.5% |
| What form of exercise is this?  Gym/fitness  Walking  Running/jogging | 43.0%  23.7%  19.3% | 42.1%  24.6%  21.9% |
| Food intake:  Average  Healthy  Unhealthy  Very healthy  Very unhealthy | 54.4%  31.6%  7.9%  5.3%  0.9% | 43.0%  43.0%  8.8%  5.3%  0.0% |
| Type of foods consumed:  Consumption of sugary items like biscuits, cakes, cream cakes, and sweet pies  Chocolate  Take-aways  Sweets/candy  Jam/honey/syrups  Sugary breakfast cereals  Chewing gum containing sugar | 86.8%  82.5%  77.2%  58.8%  67.5%  41.2%  32.5% | 86.0%  79.8%  69.3%  64.0%  52.6%  37.7%  30.7% |
| How often any of those foods are consumed: Several times weekly  Every day  Once a week  Several times a day  Several times a month  Seldom/never | 46.5%  28.1%  12.3%  7.9%  2.6%  2.6% | 45.6%  15.8%  21.9%  4.4%  11.4%  0.9% |
| Non-alcoholic drink intake:  Average  Healthy  Very healthy  Unhealthy  Very unhealthy | 34.2%  32.5%  23.7%  9.6%  0.0% | 40.4%  32.5%  14.0%  13.2%  0.0% |
| Consumption per day:  1-2 times  3-4 times  5+ times | 87.5%  12.5%  0.0% | 68.4%  21.9%  9.6% |
| Type of non-alcoholic drink intake:  Sugary soft drinks  Coffee with sugar  Tea with sugar  Milk with sugar | 52.6%  35.1%  24.6%  11.4% | 61.4%  41.2%  36.0%  16.7% |
| Energy drink consumption | 22.8% | 31.6% |
| Consumption of energy drinks per day:  1-2 drinks  3-4 drink | 100.0%  0.0% | 100.0%  0.0% |
| Changes in energy drinks consumption since starting university | 88.5% | 94.4% |
