# Supplementary material for: The association between oral health and risk behaviours of university students
Source: PLoS One. 2025 Mar 18;20(3):e0309183. doi: 10.1371/journal.pone.0309183 (PMC11918317; doi:10.1371/journal.pone.0309183)
Supplement: S5 Table — (DOCX) [file pone.0309183.s005.docx]

**Supporting information:**

**S5 Table:** Non-significant associations observed from the behaviour questionnaire over time.

| **Non-significances comparisons over time:** | **Test statistic^a^ and P value^c^** |
| --- | --- |
| Oral care routine rating | Z=-1.779, p=0.075 |
| Bleeding gums when brushed | Z=-0.572, p=0.567 |
| Experiencing toothache in the past 6 months | X^2^=0.679, p=0.410^b^ |
| Using toothpaste containing fluoride | Z=-0.366, p=0.714 |
| Importance of attending the dentist regularly | X^2^=0.973, p=0.324^b^ |
| Reason to visit the dentist | Z=-0.403, p=0.687 |
| Smoking | Z=-0.841, p=0.401 |
| Vaping | Z=-0.788, p=0.431 |
| Belief about weight (overweight, underweight etc.) | Z=-0.443, p=0.658 |
| Happiness regarding weight | X^2^=0.481, p=0.488^b^ |
| Energy drink consumption | X^2^=1.306, p=0.253^b^ |
| Energy drink frequency | X^2^=1.688, p=0.194^b^ |
| Energy drink changes over time | X^2^=2.630, p=0.105^b^ |
| Exercise | Z=-0.030, p=0.976 |
| ^a^Wilcoxon signed-rank test, unless otherwise stated, ^b^McNemar test,  ^c^Significance level is set at p<0.05 | |
